# Flexor Digitorum Longus Strain in a Spinal Cord Injury Patient: A Case Report of a Rare Diagnosis

**DOI:** 10.7759/cureus.101958

**Published:** 2026-01-21

**Authors:** Júlia Ribeiro, Daniel Cardoso, Maria Martin, André Lima, Frederico Moeda, Cristina Marques Baptista, Mónica Jorge, Diogo Portugal

**Affiliations:** 1 Serviço de Medicina Física e de Reabilitação, Hospital de Faro, Faro, PRT; 2 Serviço de Reabilitação de Adultos, Centro de Medicina e de Reabilitação de Alcoitão, Cascais, PRT; 3 Physical Medicine and Rehabilitation, Centro de Medicina de Reabilitação de Alcoitão, Lisbon, PRT; 4 Physiotherapy, Centro de Medicina e de Reabilitação de Alcoitão, Cascais, PRT; 5 Physical Medicine and Rehabilitation, Centro Hospitalar Lisboa Ocidental, Lisbon, PRT; 6 Physical Medicine and Rehabilitation, Centro de Medicina de Reabilitação de Alcoitão, Cascais, PRT

**Keywords:** calf injuries, flexor digitorum longus, muscle strain, spinal cord injury, ultrasound (u/s)

## Abstract

Calf injuries are common, and they are often described in the gastrocnemius, soleus, or plantaris muscle. They are also documented in the flexor hallucis longus and the tibialis posterior, although less frequently. We present a case of an acute flexor digitorum longus muscle strain, a rare injury, often underreported and not well-documented in the literature.

We describe the case of a 34-year-old active female undergoing rehabilitation for an incomplete tetraplegia, with suspected multiple sclerosis and left limb monoparesis. She sustained acute pain in her right leg during eccentric plantar flexion movement. Examination revealed pain with flexion of the second to fifth toes. High-resolution ultrasound confirmed disruption of flexor digitorum longus muscle fibers. The patient underwent conservative rehabilitation and returned to her pre-injury activity level within eight weeks, although subclinical strength deficits in the right limb were evident in the gait analysis.

Flexor digitorum longus strains are rare but exist and should be considered in the differential diagnosis of calf muscle injuries in healthy and disabled patients. Baseline neurological deficits can influence presentation and rehabilitation progression. Further research is needed to clarify prognosis and return-to-activity criteria for flexor digitorum longus strains.

## Introduction

The flexor digitorum longus (FDL) muscle is located in the deep posterior compartment of the leg. It originates distal to the soleal line in the middle third of the posterior aspect of the tibia, and it traverses the deep compartment of the ankle, where it becomes a tendon. The FDL tendon attaches to the plantar surface of the foot through the flexor retinaculum. It is responsible for flexion of the second, third, fourth, and fifth toes, which differentiates it from the flexor hallucis longus (FHL) - responsible for flexion of the great toe - and tibialis posterior (TP), responsible for foot inversion and plantar flexion of the ankle [1]. The FDL has some anatomical variations in relation to the FHL, the quadratus plantae, and the flexor digitorum accessorius longus [2-4].

Calf injuries are common in active people, and they are often described in the gastrocnemius, soleus, or plantaris muscle. The most common sites of injury are the gastrocnemius, preferentially involving the medial head, followed by the soleus and the plantaris [5-7]. Although less frequent, there are also reports of injuries in the FHL and the TP, both belonging to the deep posterior compartment of the leg [7-9]. A group of sports medicine experts believe that FHL strain is a more common and underreported strain than TP and FDL, although the latter can also occur and are underreported, probably due to limited use of imaging methods, especially outside sports medicine contexts. [10]. Neurological and spinal cord injury (SCI) patients can also suffer calf injuries, which are essentially described in Paralympic sports, but no FDL strain is described in this population [11].

To the best of our knowledge, no FDL strain has been described in the literature yet. This case aims to highlight the diagnostic challenges and rehabilitation implications of FDL strain in a patient with SCI.

## Case presentation

A 34-year-old female was admitted to a rehabilitation center due to an incomplete tetraplegia, AIS C (ASIA Impairment Scale) C5, under investigation since 2022. She presented a monoparesis of her left lower limb with overall strength Medical Research Council (MRC) grade 4, thermal and sensitive hypoesthesia, and proprioceptive deficit, without spasticity, walked with two walking sticks and an ankle foot orthosis (AFO) (foot-up) on the left lower limb.

Rehabilitation initially emphasized gait retraining and progressive functional tasks, advancing from supported ambulation to independent walking, outdoor adaptation, and the introduction of running and plyometric drills. To stimulate the paralyzed limb, neuromuscular electrical stimulation (NMES) was used during rehabilitation, and wet warmth was used for relaxation and recovery.

**Figure 1:**
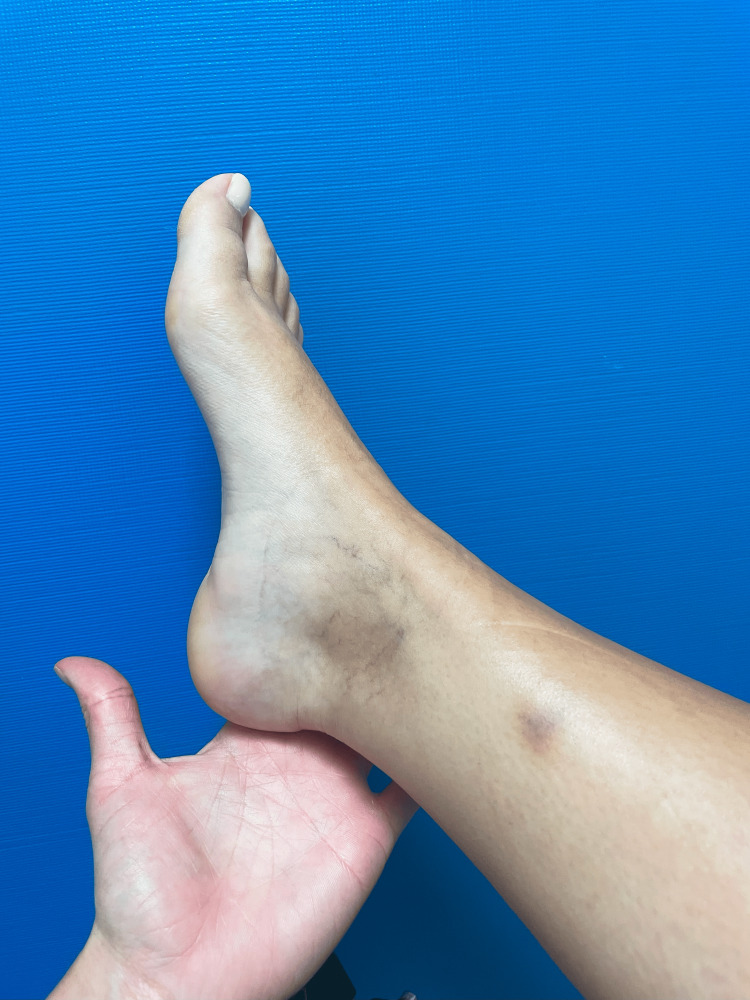
Right ankle bruise, five days after the injury

**Figure 2:**
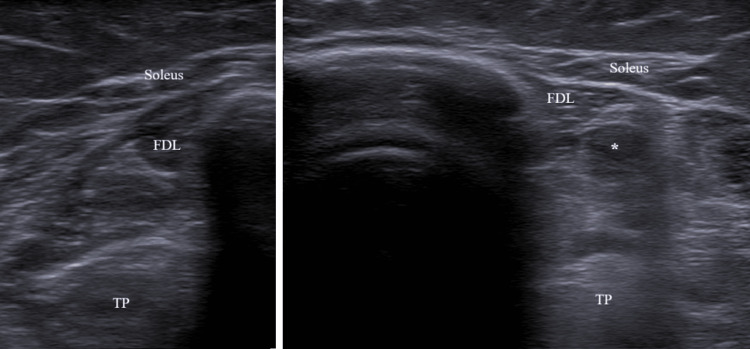
Comparison between right (muscle strain) and left flexor digitorum longus in short axis FDL: flexor digitorum longus; TP: tibialis posterior *Flexor digitorum longus muscle fiber disruption. Image captured with a Siemens ACUSON NX3TM, Siemens Medical Solutions, 5.0-10 Hz linear transducer.

**Figure 3:**
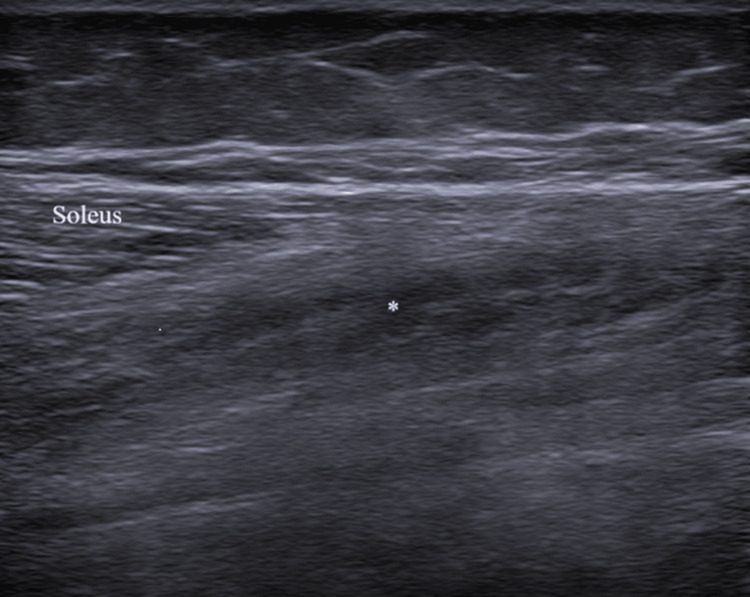
Flexor digitorum longus strain in the long axis *Flexor digitorum longus muscle fiber disruption. Image captured with a Siemens ACUSON NX3TM, Siemens Medical Solutions, 5.0-10Hz linear transducer.

During an advanced plyometric activity - when she was able to walk, run, and jump with an AFO on the left lower limb - the patient sustained a muscle rupture, which required restructuring of the program. She presented with acute severe pain in her right leg while performing an extra set of a stepping exercise. The right limb was the stepping limb, and the injury occurred during an eccentric plantar flexion movement of the ankle. She reported a sharp, sudden pain localized in the lower third of her medial leg, without preceding symptoms or a palpable defect. We examined her five days after the injury, and her gait was painful. There was tenderness medially to the Achilles tendon, mild swelling, and bruising (Figure 1). The flexion of the second, third, fourth, and fifth toes was moderately painful; the plantar flexion of the ankle in normal, adducted, and abducted positions was mildly painful; and the flexion of the first toe was not painful. Ankle and finger range of motion were intact. Due to her previous diagnosis, it was not possible to evaluate an additional strength deficit in comparison with the left limb. High-resolution ultrasound (Siemens ACUSON NX3TM, Siemens Medical Solutions, 5.0-10 Hz linear transducer) confirmed a FDL muscle strain with localized muscle fibers disruption (Figures 2-3), instead of the normal speckled appearance from hyperechoic perimysial septa amid hypoechoic fibers.

Therapeutic intervention

Her prior rehabilitation program, focused on her neurological impairment, was adapted to include a structured regimen targeting FDL strain recovery and re-injury prevention. She used an immobilization bandage during the first week to limit excessive dorsiflexion and protect the injured area. Subsequent rehabilitation prioritized proprioceptive training, pain-free strengthening, and controlled loading, beginning with a tiptoe stance and progressing to resistance exercises incorporating plantar flexion under light load. Electrotherapy and ice were used for analgesia. NMES was also employed to maintain activation of the surrounding musculature without causing undue stress to the injured structure. Walking was reintroduced at a comfortable pace without symptoms, followed by functional strengthening, including tiptoe squats and later controlled plyometric activities. No pharmacological treatment was used.

Follow-up and outcomes

Eight weeks after the injury, the patient was able to ambulate on tiptoes, perform controlled jumps without pain, and start gradual running exposure, reflecting near-complete restoration of functional capacity.

Nine weeks after the injury, a three-dimensional gait analysis was performed to compare gait with a plastic (situation 1) versus a carbon posterior left AFO (situation 2). The patient walked at a self-selected pace in a straight line across four force plates embedded into the floor (AMTI OR6-7-2000) and a baropodometric platform RsScan®. Motion data were acquired using the Vicon® Motion Capturing System. Spatiotemporal measurements as well as bilateral kinematics of the hip, knee, and ankle were studied. Her gait speed was reduced with both the plastic and the carbon AFO, due to shortened step length and decreased cadence. The shortening was influenced by the dorsiflexion deficit of the left ankle during the swing phase and the push-off deficit of the left ankle at late stance. No significant differences were observed between the two AFOs. However, with the posterior left carbon AFO, the patient displayed a slightly more symmetrical and stable gait, characterized by earlier transfer of body weight after initial contact on the left, allowing greater ankle joint power on the right. Comparing with the norm value (3.8 W/kg), the patient presented a reduced power peak in both ankles with both AFO (45% right, 15% left in situation 1, and 35% right and 12.5% left in situation 2) [12]. Although she could benefit from plastic and carbon posterior AFOs, she preferred the foot-up orthosis she had been using for a long time, and she was discharged with it.

## Discussion

Posterior calf muscles are responsible for plantar flexion, which provides the propulsive force during walking, running, or jumping. The TP, fibularis longus, and FDL also control foot pronation or supination and maintain the arches of the foot. The FDL and the TP improve the efficacy of the propulsion force transmission when they contract isometrically to maintain the height of the longitudinal arch of the foot [13].

Biomechanically, it seems that FDL are mostly activated when a heel raise is performed in adduction. In an adducted heel raise, the percentage of activation of FDL is 53.7%, whereas in a normal heel raise, it is 36.3%. Supination is mostly performed by TP, followed by FDL. The patient would further activate FDL if she had increased her weight bearing on the second to the fifth toes while performing the step. Indeed, our patient performed most of her plantar flexion movements in adduction within a closed kinetic chain, making it likely that she used this movement pattern at the time of injury [14].

The clinical findings suggested a posterior calf muscle strain. In this case, the most frequent differential diagnosis is a medial gastrocnemius or a soleus strain. However, the bruise location and the resisted tests were not in favor of these diagnoses and indicated a more probable TP or FDL strain. The strongest pain with flexion of the second, third, fourth, and fifth toes gave us a cue to the FDL strain, but we consider that, without imaging methods, it would be very difficult to differentiate an FDL from a TP strain.

High-resolution ultrasound imaging was used to confirm the diagnosis owing to its ready availability and lower cost. The scans revealed discontinuity of muscle fibers and edema of the muscle, which were consistent with an acute grade 2 muscle rupture [15]. Magnetic resonance imaging was not available, although it is the gold standard for assessing injuries in the deep posterior compartment of the leg. Nonetheless, the clinical and ultrasound findings obtained by a Physical Medicine and Rehabilitation doctor with over ten years of experience were clear enough to confirm the diagnosis [5].

Several risk factors for calf injuries are known; however, most of the high-quality studies are performed in recreational or elite athletes. In this population, the strongest intrinsic risk factors are a recent or past history of that same injury or the history of another muscle strain, which was not the case in our patient [16]. The female gender and low level of physical fitness at the beginning of a training program could have been intrinsic risk factors for injury [17].

In our case, we believe that the most important risk factors were sensory deficits in the lower limb - hypoesthesia and proprioceptive deficit - and subclinical strength deficit in the right limb (internal risk factors), with a consequent high load variation (external risk factor). Our patient is young and has high functional ambition. She had presented with a tetraplegia in the past, recovering to a strength level MRC grade 5 in the right leg. Nonetheless, this scale may be unable to discriminate between small strength deficits with an impact on higher functional activities like running or jumping. Consequently, it can be harder to periodize training to minimize high load variations. Maybe if other objective tests - isokinetic/isometric dynamometry or hand-held dynamometry - or functional tests - calf-raise test, the single-leg calf-raise test, one repetition maximum, countermovement jump - were applied, there would have been a better assessment of the readiness of our patient to run or to jump safely [18-20]. In the gait analysis, she also presented a reduced power peak in the right ankle, which could mean a subclinical strength deficit. Additionally, the injury occurred while the patient was performing an extra series of an exercise, without the physiotherapist’s guidance, which may have put her at risk of injury.

Sensory deficits in SCI patients also contribute to an increased injury risk by impairing motor control and coordination, potentially altering running and plyometric biomechanics [10,21].

Since there are no FDL strains described in the literature, the functional impairment of this injury is unknown.

Our patient underwent a conservative rehabilitation process. The acute phase focused on pain management, early mobilization, avoiding excessive dorsiflexion, and proprioceptive training. NMES facilitates the activation of the surrounding musculature while preventing excessive load on the FDL muscle. Progressive loading was subsequently initiated to restore full strength and function. This phase focused on the gradual reintroduction of eccentric and concentric calf exercises, aimed at restoring neuromuscular control while enhancing the stability of the ankle complex. Given that the FDL and TP exhibit peak activation during plantar flexion and adduction within a closed kinetic chain, it can be hypothesized that exercises incorporating this movement pattern may optimize the strength development of these muscles. However, to date, no studies have investigated the effects of exercise protocols specifically targeting the FDL [14]. In the final phase, plyometric and gradual running exposure was started, without complications.

She returned to the previous activity level in eight weeks. Given the patient’s prior physical limitations, it is challenging to compare this time frame with the time frame of strains observed in other calf muscles. For example, calf injuries in athletes take four to eight weeks of rehabilitation to return to previous activity levels, and the reported cases of TP and FHL strains took six and four weeks to return to previous activity levels, respectively [8,9,22]. More reports are needed to make conclusions regarding the return to previous activity timings.

## Conclusions

FDL muscle strain is an uncommon but important differential diagnosis in posteromedial calf pain. Ultrasound can confirm the diagnosis and support a structured, criterion-based rehabilitation program. Importantly, this case occurred in a patient with pre-existing neurologic impairment undergoing rehabilitation, highlighting that FDL strain can arise outside purely musculoskeletal or athletic contexts and that baseline neurologic deficits and assistive-device use may influence presentation and progression. Additional case reports and cohort data are needed to clarify prognosis and return‑to‑activity criteria.
